# Fear of Childbirth and Preferences for Prevention Services among Urban Pregnant Women in a Developing Country: A Multicenter, Cross-Sectional Study

**DOI:** 10.3390/ijerph18105382

**Published:** 2021-05-18

**Authors:** Lam Duc Nguyen, Long Hoang Nguyen, Ly Thi Ninh, Ha Thu Thi Nguyen, Anh Duy Nguyen, Linh Gia Vu, Cuong Tat Nguyen, Giang Thu Vu, Linh Phuong Doan, Carl A. Latkin, Cyrus S. H. Ho, Roger C. M. Ho

**Affiliations:** 1 Department of Anaesthesiology, Hanoi Medical University, Hanoi 100000, Vietnam; lamgmhs75@gmail.com; 2 Department of Global Public Health, Karolinska Institutet, SE-171 77 Stockholm, Sweden; hoang.nguyen@ki.se; 3 Social Affair Department, Ca Mau Obstetrics & Pediatrics Hospital, Ca Mau 98000, Vietnam; bachkimdoly@gmail.com; 4 Hanoi Obstetrics and Gynecology Hospital, Hanoi 100000, Vietnam; thuha.ivf@gmail.com (H.T.T.N.); bsanhbnhn@yahoo.com (A.D.N.); 5 Institute for Global Health Innovations, Duy Tan University, Da Nang 550000, Vietnam; nguyentatcuong@duytan.edu.vn (C.T.N.); doanphuonglinh.ighi@gmail.com (L.P.D.); 6 Faculty of Medicine, Duy Tan University, Da Nang 550000, Vietnam; 7 Center of Excellence in Evidence-Based Medicine, Nguyen Tat Thanh University, Ho Chi Minh City 70000, Vietnam; giang.coentt@gmail.com; 8 Bloomberg School of Public Health, Johns Hopkins University, Baltimore, MD 21205, USA; carl.latkin@jhu.edu; 9 Department of Psychological Medicine, Yong Loo Lin School of Medicine, National University of Singapore, Singapore 119077, Singapore; cyrushosh@gmail.com (C.S.H.H.); pcmrhcm@nus.edu.sg (R.C.M.H.); 10 Department of Psychological Medicine, National University Health System, Singapore 119228, Singapore; 11 Institute for Health Innovation and Technology (iHealthtech), National University of Singapore, Singapore 119077, Singapore; 12 Institute of Health Economics and Technology, Hanoi 100000, Vietnam

**Keywords:** fear, childbirth, pregnant women, prevention, preferences

## Abstract

This study aimed to examine fear of childbirth and willingness to pay for fear-prevention services in pregnant women. A multicenter, cross-sectional study was conducted on pregnant women in two obstetric hospitals in Vietnam. The Fear of Birth Scale was utilized to evaluate fear of childbirth. Multivariable, generalized linear regression and logistic regression models were performed to identify associated factors with fear of childbirth, demand, and willingness to pay for prevention services. Of 900 pregnant women, fear of childbirth was moderately high with a mean score of 18.1 (SD = 2.3). Age of partner; ever having complications of pregnancy; attitudes toward different aspects of childbirth delivery; satisfactions with friends, parents, and siblings’ care; and information support were associated with fear of childbirth. Only 33.8% participants had a demand for the prevention service, and 43.7% were willing to pay for this service with an average amount of $US 10.0 per month (SD = 72.0). Our study suggested that individualized psychological counseling and information-seeking guidance should be provided appropriately and differently for multiparous and nulliparous women for reducing fear and improving the acceptability of the prevention services.

## 1. Introduction

Tokophobia, or fear of childbirth, is a common psychological health problem in pregnant women, ranging from normal condition to extreme fear or pathological fear [1]. This issue is a socio-cultural health problem because women can develop fear as a result of observing other people’s birth experiences (e.g., relatives or friends) [2,3]. Moreover, it can be related to women’s pregnancy and birth expectancy, increasing the risk of anxiety disorders and then, in turn, influencing development of fear [4]. Other reasons for fear can be their prior experience with sexual abuse or trauma in their childhood, leading to their avoidance of pregnancy and childbirth even though they desire a child [5].

Fear of childbirth increases the risk of various physical problems. Such as sleep disturbances, stomach pain, limited daily activities [6,7,8,9], prolonged birth duration as well as the risk of abortion, miscarriage, or stillborn infant [10]. It is also associated with different negative psychological and social consequences like postnatal depression, emotional imbalance, and difficulties in partner and mother-child relationships. Fear of childbirth can have long-term impacts when some women cannot overcome the fear and decide to be childless [10]. Furthermore, fear of childbirth is associated with high-risk interventions, such as cesarean surgery or overuse of epidural anesthesia to relieve pain, which might put pregnant women in danger if insufficient pain control strategies are implemented [11,12,13]. Although adverse impacts of fear of childbirth are significant, information about services for childbirth-related fear prevention and management among pregnant women is lacking in both developed and developing countries [14]. Evidence from Western countries, such as Sweden and England, indicated that the majority of obstetric clinics did not offer special services for childbirth fear, and the authors called actions to standardize the procedure of care and treatment for women with childbirth fear [15,16].

In Vietnam, the rate of cesarean section rapidly increased from 10% in 2002 to 28% in 2014 [17], and fear of childbirth is considered a major attributable factor [18]. Limited knowledge about fear of childbirth and associated factors, as well as preferences for fear-prevention services in this population, is available. This multicenter study aimed to examine fear of childbirth and willingness to pay for fear-prevention services in pregnant women.

## 2. Materials and Methods

### 2.1. Study Design and Procedures

From January to February 2021, a multi-site, cross-sectional study was performed at two hospitals in two cities in Vietnam: Hanoi and Ca Mau. Eligible criteria included: pregnant women; 18 years or above; willing to participate in the study; and gave signature for informed consent. Pregnant women were excluded if (1) they had chronic conditions, cognitive impairment, or other conditions which might affect their capacity to respond to the interview; and (2) pregnant women having prior miscarriages or stillborn baby. Women who did not complete the interview were also excluded. A convenient sampling procedure was used to recruit participants. We invited all eligible pregnant women visiting the hospitals for regular antenatal care or baby delivery during the study period. We approached the former group (antenatal care) when they had completed all required examinations whereas we met the latter group (baby delivery) two or three days after delivery. Participants were face-to-face interviewed by trained data collectors who were nurses or medical students for 15–20 min. We developed a self-reported structured questionnaire and then piloted it among 10 pregnant women with different gestation weeks. The questionnaire was modified according to their comments to ensure that its contents were clear and understandable. Pregnant women were screened by our physicians to ensure that they met the eligible criteria, then eligible participants were invited to a private room for confidentiality. They were informed about the study’s purposes, their benefits, and their rights when participating in the study. They were also informed that they could withdraw at any time from the interview, and their withdrawal did not affect any services that they received. After that, we obtained their signed informed consent and started to interview. No incentive was provided to the participant. A total of 1019 pregnant women met our inclusion criteria and were invited and enrolled. We excluded data of 119 pregnant women who did not respond to the questions about fear of childbirth (completion rate 88.3%), resulting in the final sample size of 900 pregnant women. Differences between excluded and included women in age (mean = 28.8 years vs. mean = 28.2 years, *p* = 0.33, respectively), number of children (83.3% having children vs. 72.4%, *p* = 0.19, respectively), frequency of antenatal care (88.0% having care once a month or less vs. 91.1%, *p* = 0.06, respectively), and having any pregnancy complications (10.9% vs. 14.3%, *p* = 0.313, respectively) were not statistically significant.

### 2.2. Variables

Fear of childbirth: We adapted a six-item scale called Fear of Birth Scale, which was published elsewhere, to measure levels of fear of childbirth among pregnant women [19,20,21]. This instrument was developed and adopted from the Wijma Delivery Expectancy/Experience Questionnaire, which is the most common instrument for childbirth fear screening [8]. Each item had five levels of response: 1 = Strongly disagree; 2 = Disagree; 3 = Neutral; 4 = Agree; 5 = Strongly agree. The score of the last three items had to be reversed. The total score ranged from 6 to 30, in which a higher score indicated a higher level of childbirth fear. The internal consistency reliability of this scale was acceptable with a Cronbach’s alpha of 0.7597.

Preferences for fear-prevention services: First, we described the proposed prevention services that could include the following non-pharmacological components aiming to protect pregnant women from fearing childbirth: psychological counseling, health education in terms of physical activity/meditation, and hypnosis-based therapy. These services were selected according to a previous systematic review [22]. Then, we asked participants to report whether they had a demand for fear-prevention services, their preferred location to receive services, and service providers. Furthermore, we asked whether they would be willing to pay for these services and the amount of the willingness to pay.

Attitudes toward aspects of childbirth delivery:

Concerns about physical changes: We used a four-item scale to measure the concerns about physical changes or body image changes including [19]: (1) I am worried about the physical changes that occur in a woman’s body during pregnancy; (2) I am worried about the physical changes that occur in a woman’s body after pregnancy; (3) I am afraid of what the labor and delivery process will do to my body; (4) changes that might occur to a woman’s perineal (pelvic) floor after a vaginal birth are a concern for me [19]. There were five response options from 1 = Strongly disagree; 2 = Disagree; 3 = Neutral; 4 = Agree; 5 = Strongly agree. The total score ranged from 4 to 20, in which a higher score indicated a higher level of concerns about physical change. The Cronbach’s alpha was 0.8334.

Attitude toward childbirth delivery preparation: Regarding childbirth delivery preparation, we asked participants to express their agreement (from 1, “Strongly disagree”, to 5, “Strongly agree”) with different statements about their attitudes toward cesarean surgery (i.e., “I do not worry about cesarean surgery”), technology use in obstetric care (i.e., “Using advanced technology in child delivery is necessary”), confidence about pregnancy-related knowledge (i.e., “I am confident in my knowledge of childbirth and reproductive health care”), and ease to seek information about maternal care (i.e., “I can easily find information regarding birthing and reproductive health services”). The higher scores indicated a higher level of a positive attitude about different aspects of childbirth delivery preparation.

Expectancy of having a baby, perception about risky pregnancy, and pain of childbirth delivery: Expectancy of childbirth was evaluated by rating a single item “To what extent were you expecting to have a baby?” from 0, “Totally unexpected”, to 10, “Totally expected”. Meanwhile, we measured the perceptions about the risk of pregnancy by asking them the question, “How risky do you think it is to give birth?” and participants rated their answer from 0, “Absolutely free of risk”, to 10, “Extreme risky”. In addition, we asked participants to rate the following statement: “How much pain do you associate with giving birth?” from 0, “Completely painless”, to 10, “Extreme pain”.

Social support: We employed the Perinatal Infant Care Social Support Scale (PICSS) to assess social support for maternal care [23]. This instrument had 22 items with four options for each item (from 1, “Strongly disagree”, to 4, “Strongly agree”) to evaluate four domains of functional social support, including information support (7 items–range score from 7 to 28), instrument support (7 items–range score from 7 to 35), emotional support (4 items–range score from 4 to 16), and appraisal support (4 items–range score from 4 to 16). A higher score indicated a higher level of social support. The Cronbach’s alpha was excellent at 0.9742. Moreover, we asked pregnant women to rate their satisfaction (from 0, “Completely dissatisfied”, to 10, “Completely satisfied) with different people’s care during their pregnancy, including partner, parents-in-law, parents, siblings, relatives, and friends.

Sociodemographic and maternal characteristics: Data on sociodemographic and maternal characteristics included location (Hanoi/Ca Mau), age, education, partner’s age, partner’s education, parity status (nulliparous or multiparous), number of children, pregnancy status (being pregnant or recently delivered), frequency of antenatal care visit, preferable delivery method (vaginal/cesarean method), information sources for maternal care, and whether they had experienced any complications of pregnancy during the pregnancy period.

### 2.3. Statistical Analysis

Descriptive data analysis was performed using Chi-squared and Mann–Whitney tests to assess the differences of study variables between nulliparous and multiparous pregnant women. We used multivariable, generalized linear regression models to identify associated factors with fear of childbirth in these two groups of pregnant women. Meanwhile, we applied multivariable, logistic regression models to determine factors related to the demand for prevention service and willingness to pay for the service. A stepwise strategy using the backward selection method with a *p*-value of log-likelihood test for including variables of 0.2 was utilized along with the regression models to identify optimal models. The significance level was set at a *p*-value < 0.05. Stata software version 16.0 (StataCorp LLC, Texas, USA) was used for data analysis.

## 3. Results

Table 1 summarizes sociodemographic and maternal characteristics of respondents. Of 900 pregnant women, most of them were multiparous (72.4%), and 44.2% recently delivered an infant before the interview. The mean age of the sample was 28.2 (SD = 5.2) years old, and the mean age of their partners was 31.1 (SD = 5.6) years old. We found significant differences in age, partner’s age, number of children, pregnancy status, frequency of antenatal care visit, and preferable delivery method between nulliparous and multiparous pregnant women (*p* < 0.05).

Table 2 depicts that the fear of childbirth in our sample was moderately high with a mean score of 18.1 (SD = 2.3, range score 7–26), and no difference was found between nulliparous and multiparous pregnant women (*p* = 0.81). Multiparous women had a significantly higher level of confidence about pregnancy-related knowledge (mean = 3.4, SD = 0.7) compared to nulliparous women (mean = 3.1, SD = 0.7) (*p* < 0.01). Meanwhile, no differences regarding other factors such as concerns about physical changes, worry about cesarean surgery, attitude toward technology, information seeking, expectancy of having a baby, and perceived risk of pregnancy were found between both groups (*p* > 0.0).

Results of multivariable regression analysis are shown in Table 3. Only variables included in the final models by stepwise selection strategy are presented. Among multiparous women, a higher age of partner (Coef. = −0.10; 95%CI = −0.16; −0.05), a higher level of attitude toward ease of seeking information (Coef. = −0.42, 95%CI = −0.78; −0.07), and a higher level of satisfaction with parents’ care (Coef. = −0.38; 95%CI = −0.59; −0.16) were associated with a lower score of fear of childbirth. Meanwhile, women who recently delivered a baby, ever experiences complications of pregnancy, had higher scores of concerns about physical changes and perceived risk of pregnancy, and had a higher level of satisfaction with siblings’ care were related to a higher score of fear of childbirth.

In nulliparous women, having friends/relatives as a primary source of maternal care information (Coef. = −0.83, 95%CI = −1.42; −0.23) and a higher level of confidence about pregnancy-related knowledge (Coef. = −1.04, 95%CI = −1.51; −0.58) significantly reduced the score of fear of childbirth. In contrast, receiving information from health professionals, having a higher attitude towards ease of seeking information, expected pain of childbirth delivery and concerns about physical changes, higher level of satisfaction with friends, and a higher level of information support could significantly increase the fear of childbirth in nulliparous women.

Table 4 shows no difference in the preferences for fear of childbirth prevention services between nulliparous and multiparous women (*p* < 0.05). Only a third of participants had a demand for this service (33.8%), and 43.7% were willing to pay for this service with an average amount of $US 10.0 (SD = 72.0). Among those having demand for the service, the majority of them preferred to receive the service at the hospital (69.1%), which was provided by the physicians (83.6%).

Factors associated with demand and willingness to pay for fear-prevention service are presented in Table 5. Pregnant women who visited antenatal care clinics less than once per month were more likely to have demand for the service (OR = 3.05, 95%C = 1.26; 7.43) compared to those visiting once per week. Meanwhile, higher satisfaction with friends’ care was negatively associated with demand for the service. People with cesarean as the preferred delivery method and those with higher satisfaction with parents’ care were also more likely to have demand for the service as well as be willing to pay for the service; however, receiving maternal care information from radio/television reduced the likelihood of having demand for the service (OR = 0.46, 95%CI = 0.27; 0.79) and being willing to pay for the service (OR = 0.43, 95%CI = 0.25; 0.73). Other factors, such as higher education, receiving information from newspapers/magazines, and ever having complications of pregnancy, were associated with a higher likelihood of being willing to pay for the service.

## 4. Discussion

Fear of childbirth can be normally considered a common phenomenon, given the risk and painfulness of pregnancy and the childbirth period. The findings of this study revealed that the majority of pregnant women in our sample had a moderate level of childbirth fear, which was similar to results from previous studies [11,12,14,24,25,26]. Notably, we did not find any association between parity and fear of childbirth, which is contradicting to prior findings [8,14,27]. However, our result was similar to the results of a longitudinal cohort in Finland, which showed that higher fear of childbirth was observed in multiparous women compared to that in nulliparous women [28]. The author explained that negative prior experience could be the reason for this association [28]. Unfortunately, information about previous childbirth experiences was not available in our study, suggesting further studies to address this knowledge gap.

Factors associated with fear of childbirth have been reported in many studies, including low socioeconomic characteristics, pregnancy status, the preferred mode of delivery, poor health conditions, and low social support [8,27]. Our research found no difference in childbirth fear between nulliparous and multiparous women, which was different from other previous studies [8,19,24,28]. This difference could be justified by the variance of demographic and maternal characteristics across studies. Furthermore, given the high risk of pregnancy and childbirth regardless of parity status, multiparous women perhaps suffered from similar levels of fear as nulliparous ones although they had prior pregnancy experience, suggesting that interventions to address fear of childbirth should be provided to both groups of pregnancy.

In nulliparous women, we found a significant relationship between fear of childbirth and concerns about pregnancy-related knowledge, body changes, and pain during childbirth. Nulliparous women had no previous experience in pregnancy and childbirth; hence, good preparation with adequate knowledge helped them be confident during pregnancy, thereby reducing the fear of childbirth [29]. On the other hand, pregnancy and childbirth are painful processes and significantly affect the physical health of a pregnant woman. While the connection between anxiety about pain and childbirth fear was understandable [19,20], there are several reasons explaining the association between concerns about body changes and fear of childbirth. First, in the modern context, there is emphasis on keeping a beautiful and ideal body image after giving birth, as the female body illustrated in social media has put great pressure on pregnant women [30]. Second, in some women, getting pregnant can affect their physical health, especially the pelvic floor area, urinary incontinence, and sexual function [20]. The above two reasons increase the anxiety of women during pregnancy and childbirth, thereby elevating their fear of childbirth.

Noticeably, we identified the important role of information support and information-seeking behaviors in shaping childbirth fear in the nulliparous group. The PICSS defined information support as the availability of advice, knowledge, or directives women need when pregnant [23]. Previous studies showed that lower information support increased the risk of childbirth fear [31], and pregnant women were more likely to gain more information to ensure that they had sufficient preparation to prevent complications or for birth [32]. However, being overwhelmed with too much information, particularly conflicting information, could elevate childbirth fear [33,34,35]. In the past, books, friends/relatives, and health professionals were the main sources of information [34]; however, in the digital era, it is easier to access information and connect to other mothers for advice in a short time [36], but it also raises the risk of exposure to fake or conflicting information. Therefore, healthcare professionals should guide pregnant women to seek reliable information sources along with sufficient and timely emotional support to diminish their anxiety during the pregnancy period [37]. Future research examining associations between information sources and fear of childbirth should also be elucidated.

Meanwhile, multiparous women are individuals having previous childbirth experience; thus, the pattern of determinants of childbirth fear was different compared to nulliparous women. First, perceived ease in seeking information becomes their advantage in reducing fear of childbirth since these women could identify appropriate information sources for their pregnancy based on their previous experience. Second, because they had an awareness of childbirth delivery, they had more understanding about the risk of pregnancy and childbirth as well as the dangers of pregnancy complications to their health. Therefore, fear of childbirth increased proportionately with their perceived risk of pregnancy and if they ever suffered from complications. Parents’ care is vital to reduce the fear, suggesting that further fear-reduction interventions in this group should involve the role of parents as the main component.

Our study explored demands and willingness of pregnant women to pay for prevention and management services in relation to fear of childbirth. Findings indicated that the majority of participants did not know whether they could ask for these services. In addition to pregnant women expressing that they could control their fears due to their adequate preparation, we observed that a majority of the other women questioned the effectiveness of the service. In this study, we gave a hypothetical scenario that we would provide non-pharmacological interventions, including psychological counseling, health education in terms of physical activity/meditation, and hypnosis-based therapy. Currently, according to our knowledge, there is no specific service in Vietnam for women with fear of childbirth. Thus, information about these services has not been disseminated widely. Nonetheless, results implied that if women were well-informed with sufficient information for their decisions, the proportion of women having demand for the service might increase, particularly those preferring cesarean surgery and those having pregnancy complications, which can improve the feasibility and acceptability of the services. Additionally, among women who were willing to pay for the service, the average amount they were willing to pay was US$ 10, which was equivalent to the cost of an antenatal care visit. Thus, the services can be integrated with the current antenatal care model to provide comprehensive services for pregnant women as well as reduce operating costs.

Results of this study suggested several clinical and public health implications. First, previous trials showed that childbirth fear could be reduced with low-cost interventions and support from health professionals [22]; thus, it is important for pregnant women to recognize their fear and seek appropriate services to manage and control this problem. This also requires obstetric physicians to screen the fear via validated instruments, such as the Fear of Birth Scale or the Wijma Delivery Expectancy/Experience Questionnaire, when performing regular examinations. Second, given that nulliparous and multiparous women had different risk factors for childbirth fear, interventions should also be individualized for each group to maximize their effectiveness. For example, while the fear of multiparous women was associated with concerns about physical changes and risk of pregnancy, the fear of nulliparous women was related to issues regarding physical changes, information source, and pain during labor. Physicians can discuss response options for each concern in antenatal appointments, refer pregnant women to appropriate services (e.g., exercise classes or clinics that specialize in the pelvic floor), and commit to offering timely support when necessary [38]. Third, health-education programs should be implemented to raise awareness and knowledge of pregnant women about pregnancy care and the childbirth process. Information should be from reliable sources, and the message should be simple, clear, and concise. Moreover, for nulliparous women who have no prior experience with childbirth delivery, offering information should come along with psychological counseling sessions to help to reduce their anxiety and fear. Fourth, given the fact that women were not sure about the possibility of asking for the prevention service because of lacking information about the effectiveness of the interventions, health communication and education should be performed widely to raise their awareness about this service. Further studies should also be warranted to examine which interventions should be included in the service (for example peer educators) and the optimal manners of organizing the service in the hospitals.

The study has several limitations that need to be addressed. First, the design of the cross-sectional study does not allow us to evaluate the causal relationship between fear of childbirth and related factors. Further longitudinal studies should be performed to assess which factors influence the increase or decrease in fear of childbirth. Second, the cut-off point of the scale has not been determined; hence, we failed to assess the prevalence of fear of childbirth in our sample but only examined the associations between fear of childbirth score and certain key variables. Further validation studies should be conducted to find the optimal cut-off point of the scale especially when compared with other scales, such as the Childbirth Attitude Questionnaire scale (CAQ) or Wijma Delivery Expectancy Questionnaire (W-DEQ), as well as depression and anxiety scales. Third, although our study had a large sample size and was performed in several hospitals, the convenient sampling method limited our ability to generalize results to groups of pregnant women in other locations, like mountainous or rural settings. Therefore, one should be cautious when using our results for those locations. Finally, the study collected self-reported information from pregnant women, which might lead to recall bias. We attempted to minimize this bias by using proxy questions during the interview to help women recall information.

## 5. Conclusions

Our study revealed a moderate level of childbirth fear among pregnant women and the potential feasibility of fear-prevention services in some obstetric hospitals in Vietnam. Individualized psychological counseling and information-seeking guidance should be provided appropriately and differently for multiparous and nulliparous women for reducing fear and improving the acceptability of the prevention services.

## Figures and Tables

**Table 1:** Sociodemographic and maternal characteristics.

Characteristics	Total		Nulliparous		Multiparous		*p*-Value
	*n*	%	*n*	%	*n*	%	
Total	900	100.0	248	27.6	652	72.4	
Location							
Hanoi	522	58.0	142	57.3	380	58.3	0.78
Ca Mau	378	42.0	106	42.7	272	41.7	
Education							
Below high school	133	14.8	37	15.0	96	14.8	0.29
High school	290	32.3	80	32.4	210	32.3	
Colleague/vocational training	149	16.6	32	13.0	117	18.0	
Undergraduate/post-graduate	326	36.3	98	39.7	228	35.0	
Partner’s education							
Below high school	76	8.5	17	6.9	59	9.1	0.33
High school	229	25.6	63	25.4	166	25.6	
Colleague/vocational training	278	31.0	71	28.6	207	31.9	
Undergraduate/post-graduate	313	34.9	97	39.1	216	33.3	
Number of children							
None	248	27.6	248	100.0	0	0.0	<0.01
One	411	45.7	0	0.0	411	63.0	
Two or more	241	26.8	0	0.0	241	37.0	
Pregnancy status							
Pregnant women	608	67.7	245	98.8	363	55.9	<0.01
Recently delivered	290	32.3	3	1.2	287	44.2	
Frequency of antenatal care visit							
Once a week	80	8.9	34	13.7	46	7.1	<0.01
Once a month	222	24.7	44	17.7	178	27.4	
Less than once per month	72	8.0	12	4.8	60	9.2	
Less than once per three months	89	9.9	22	8.9	67	10.3	
Follow physician’s instructions	435	48.4	136	54.8	299	46.0	
Preferable delivery method							
Vaginal delivery	451	53.1	177	77.6	274	44.1	<0.01
Cesarean surgery	399	46.9	51	22.4	348	56.0	
Information sources for maternal care							
Friends/relatives	440	48.9	153	61.7	287	44.0	<0.01
Banner/poster	40	4.4	13	5.2	27	4.1	0.47
Internet/social network site	567	63.0	158	63.7	409	62.7	0.79
Mobile phone message	61	6.8	16	6.5	45	6.9	0.81
Radio, television	204	22.7	55	22.2	149	22.9	0.83
Newspaper, magazine	162	18.0	47	19.0	115	17.6	0.65
Health professionals	575	63.9	162	65.3	413	63.3	0.58
Smartphone applications	196	21.8	68	27.4	128	19.6	0.01
Others	8	0.9	4	1.6	4	0.6	0.15
Ever having complications of pregnancy	129	14.3	42	16.9	87	13.3	0.17
	Mean	SD	Mean	SD	Mean	SD	
Age (years)	28.2	5.2	24.9	4.4	29.4	4.9	<0.01
Age of partner (years)	31.1	5.6	28.2	4.9	32.2	5.5	<0.01
Satisfaction with care from (0–10)							
Partner	8.1	2.2	8.1	2.2	8.2	2.2	0.32
Parents-in-law	7.8	2.4	7.7	2.4	7.9	2.5	0.19
Parents	8.2	2.2	8.2	2.2	8.3	2.2	0.22
Siblings	8.0	2.3	7.9	2.3	8.1	2.3	0.18
Relative	8.0	2.2	7.9	2.2	8.0	2.3	0.13
Friends	7.9	2.3	7.8	2.3	8.0	2.3	0.21
Perinatal infant care social support							
Appraisal support (4–16)	12.2	1.4	12.4	1.2	12.2	1.4	0.23
Emotional support (4–16)	12.3	1.5	12.4	1.4	12.2	1.5	0.15
Information support (7–28)	21.5	2.7	21.7	2.6	21.5	2.7	0.78
Instrument support (7–28)	21.4	2.6	21.7	2.6	21.3	2.6	0.21

**Table 2:** Fear of childbirth and attitudes toward different birth aspects.

Characteristics	Total		Nulliparous		Multiparous		*p*-Value
	Mean	SD	Mean	SD	Mean	SD	
Fear of childbirth (6–30)	18.1	2.3	18.1	1.9	18.1	2.4	0.81
Concerns about physical changes (4–20)	13.2	2.4	13.3	2.3	13.2	2.4	0.42
Not worry about cesarean surgery (1–5)	3.0	0.8	3.0	0.7	3.0	0.9	0.72
Positive attitude towards technology in obstetric care (1–5)	3.7	0.8	3.7	0.8	3.7	0.8	0.64
Ease of seeking information (1–5)	3.6	0.7	3.5	0.7	3.6	0.7	0.23
Confidence about pregnancy-related knowledge (1–5)	3.3	0.7	3.1	0.7	3.4	0.7	<0.01
Expectancy of having a baby (0–10)	7.8	2.2	7.9	2.1	7.8	2.2	0.43
Perceived risky of pregnancy (0–10)	6.0	2.3	5.8	2.2	6.1	2.4	0.18
Expected pain of childbirth delivery (0–10)	6.8	2.3	6.9	2.3	6.8	2.4	0.50

**Table 3:** Factors associated with fear of childbirth between nulliparous and multiparous pregnant women.

Characteristics	Multiparous		Nulliparous	
	aCoef.	95%CI	aCoef.	95%CI
SOCIODEMOGRAPHIC CHARACTERISTICS				
Age (per year)	0.06 *	−0.00; 0.12		
Age of partner (per year)	−0.10 ***	−0.16; −0.05		
Partner’s education (vs. below high school—ref)				
High school			−1.02 *	−2.07; 0.04
Colleague/vocational training			−0.47	−1.45; 0.52
Undergraduate/post-graduate			−0.48	−1.54; 0.57
MATERNAL CHARACTERISTICS				
Information sources for maternal care				
Friends/relatives (yes vs. no—ref)			−0.83 ***	−1.42; −0.23
Banner/poster (yes vs. no—ref)			−1.16	−3.02; 0.70
Mobile phone message (yes vs. no—ref)	−0.74 *	−1.60; 0.12		
Health professionals (yes vs. no—ref)			0.65 **	0.02; 1.28
Others (yes vs. no—ref)	−2.23 *	−4.63; 0.17	−3.53 ***	−5.79; −1.27
Preferable delivery method (cesarean vs. vaginal delivery—ref)	0.35 *	−0.05; 0.76		
Pregnancy status (recently delivered vs. pregnant—ref)	0.50 **	0.10; 0.91		
Ever having complications of pregnancy (yes vs. no—ref)	0.60 **	0.04; 1.16		
ATTITUDES TOWARD PREGNANCY				
Ease of seeking information (per score)	−0.42 **	−0.78; −0.07	0.75 **	0.17; 1.32
Confidence about pregnancy-related knowledge (per score)	−0.32 *	−0.69; 0.04	−1.04 ***	−1.51; −0.58
Expected pain of childbirth delivery (per score)	0.08	−0.04; 0.19	0.27 ***	0.12; 0.41
Concerns about physical changes (per score)	0.15 ***	0.07; 0.24	0.20 ***	0.06; 0.34
Perceived risk of pregnancy (per score)	0.27 ***	0.17; 0.37		
Positive attitude towards technology in obstetric care (per score)			−0.42 *	−0.88; 0.04
SOCIAL SUPPORT				
Satisfaction with care from				
Friends (per score)			0.33 **	0.04; 0.63
Parents (per score)	−0.38 ***	−0.59; −0.16		
Partner (per score)	0.14 *	−0.03; 0.31		
Relatives (per score)			−0.28 *	−0.61; 0.04
Sibling (per score)	0.28 ***	0.07; 0.49		
Perinatal infant care social support				
Appraisal support (per score)	0.10	−0.03; 0.23	−0.27 *	−0.56; 0.02
Information support (per score)			0.13 **	0.02; 0.25

Abbrev: aCoef., adjusted coefficient; CI, confidence interval; ref, reference group. * *p* < 0.1; ** *p* < 0.05; *** *p* < 0.01.

**Table 4:** Preferences for fear of childbirth prevention services.

Characteristics	Total		Nulliparous		Multiparous		*p*-Value
	*n*	%	*n*	%	*n*	%	
Demand for prevention service (*n* = 900)							
Yes	304	33.8	78	31.5	226	34.7	0.48
No	179	19.9	47	18.9	132	20.3	
Don’t know	417	46.3	123	49.6	294	45.1	
Preferable location to receive service (*n* = 304)							
Hospital	210	69.1	51	65.4	159	70.4	0.41
Home	91	29.9	28	35.9	63	27.9	0.18
Others	15	4.9	3	3..9	12	5.3	0.61
Preferable service providers (*n* = 304)							
Physicians	254	83.6	67	85.9	187	82.7	0.52
Nurses	40	13.2	9	11.5	31	13.7	0.62
Specialists	76	25.0	19	24.4	57	25.2	0.88
Others	6	2.0	1	1.3	5	2.2	0.61
Willingness to pay for prevention service (*n* = 900)							
No	507	56.3	140	56.5	367	56.3	0.97
Yes	393	43.7	108	43.6	285	43.7	
	Mean	SD	Mean	SD	Mean	SD	
Amount of willingness to pay per month ($US) (*n* = 393)	10.0	72.0	3.3	5.4	12.3	83.4	0.84

**Table 5:** Factors associated with demand and willingness to pay for fear-prevention service.

Characteristics	Demand for Prevention Service		Willingness to Pay for Prevention Service	
	aOR	95%CI	aOR	95%CI
SOCIODEMOGRAPHIC CHARACTERISTICS				
Education (vs below high school—ref)				
High school	0.64	0.26; 1.62	1.34	0.73; 2.47
Colleague/vocational training	1.45	0.55; 3.83	2.22 **	1.10; 4.47
Undergraduate/post-graduate	1.64	0.62; 4.35	4.11 ***	2.18; 7.72
Partner’s education (vs below high school—ref)				
High school	1.70	0.62; 4.69		
Colleague/vocational training	1.45	0.53; 3.98		
Undergraduate/post-graduate	2.49 *	0.89; 7.00		
MATERNAL CHARACTERISTICS				
Frequency of antenatal care visit (vs. once a week—ref)				
Once a month	1.68	0.84; 3.34	1.50	0.73; 3.09
Less than once per month	3.05 **	1.26; 7.43	1.45	0.61; 3.44
Less than once per three months	0.56	0.13; 2.38	1.42	0.58; 3.47
Follow physician’s instructions	0.78	0.39; 1.56	0.46 **	0.23; 0.93
Information sources for maternal care				
Internet/social network sites (yes vs. no—ref)	1.61 *	0.92; 2.82		
Radio/television (yes vs. no—ref)	0.46 ***	0.27; 0.79	0.43 ***	0.25; 0.73
Newspaper/magazine (yes vs. no—ref)			1.95 **	1.12; 3.39
Preferable delivery method (cesarean vs. vaginal delivery—ref)	2.07 ***	1.35; 3.16	1.59 **	1.09; 2.32
Having complications of pregnancy (yes vs. no—ref)			1.92 **	1.12; 3.29
Fear of childbirth	1.06	0.98; 1.15		
SOCIAL SUPPORT				
Satisfaction with care from				
Friends (per score)	0.82 **	0.69; 0.99		
Parents (per score)	1.94 ***	1.52; 2.47	1.38 ***	1.13; 1.69
Relatives (per score)			0.87	0.72; 1.06
PICSS Emotional support (per score)			1.11	0.98; 1.26

Abbrev: aOR, adjusted odds ratio; CI, confidence interval; ref, reference group. * *p* < 0.1; ** *p* < 0.05; *** *p* < 0.01.

## Data Availability

The dataset of this study is available upon reasonable request.
